# Predictive Ability of the CHA2DS2‐VA Score for No‐Reflow Phenomenon in ST‐Elevation Myocardial Infarction Patients Undergoing Primary PCI

**DOI:** 10.1002/clc.70439

**Published:** 2026-08-05

**Authors:** Ying Sun, Jian Ren, Wei Wang, Chunsong Wang, Min Liu, Jiahui Liu, Xuewen Qi, Hengchen Yao

**Affiliations:** ^1^ Department of Cardiology Liaocheng People's Hospital Affiliated to Shandong First Medical University Liaocheng Shandong People's Republic of China; ^2^ Department of Cardiology, Liaocheng Dongchangfu People's Hospital Liaocheng People's Hospital Dongchangfu Campus Liaocheng Shandong People's Republic of China; ^3^ Liaocheng People's Hospital Shandong University Jinan Shandong People's Republic of China

**Keywords:** Acute myocardial infarction, CHA2DS2‐VA score, no‐reflow phenomenon, sex difference, ST‐elevation myocardial infarction

## Abstract

**Background:**

No‐reflow phenomenon (NRP) frequently complicates ST‐elevation myocardial infarction (STEMI). This study evaluated the CHA_2_DS_2_‐VA score (CV score) as a predictor of NRP in STEMI patients undergoing primary PCI.

**Methods:**

A total of 725 STEMI patients were retrospectively enrolled and stratified by median of CV score (≤ 2 vs. > 2). The predictive ability of the CV score for NRP was assessed using logistic regression, receiver operating characteristic analysis, and DeLong tests.

**Results:**

NRP incidence was higher in the high CV score group (15.2% vs. 8.7%, *p* = 0.007). The CV score predicted NRP overall (adjusted OR: 1.295, *p* = 0.002), but this effect was confined to females (AOR: 1.597, *p* = 0.002), with no significant association in males. Area under the curve value (AUC) of the CV score for predicting NRP was 0.613 in all patients (38.8% sensitivity and 79.1% specificity, *p* = 0.001) and 0.673 in females (78.1% sensitivity and 47.6% specificity, *p* = 0.002). A significant sex × CV score interaction (*p* = 0.017) confirmed sex‐modified predictive value. DeLong tests combining the CV score with laboratory markers improved prediction in males but not females.

**Conclusion:**

The CV score is a sex‐specific predictor of NRP in STEMI, with predictive value in females but not males. These findings support a tailored risk stratification approach: using the CV score alone for female patients, while integrating laboratory and procedural markers for male patients.

## Graphical abstract

The CHA_2_DS_2_‐VA score predicts no‐reflow after primary PCI in female STEMI patients (AUC 0.673) but not males. These sex‐specific findings support tailored risk stratification: standalone CV score use for women, versus integration with laboratory and procedural markers for men.

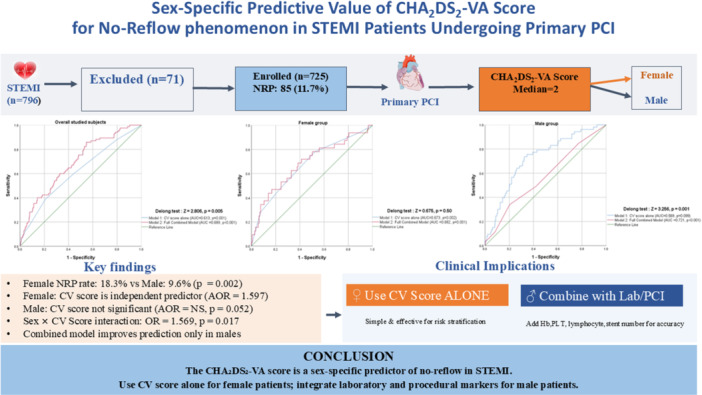

## 1 Introduction

With the improvement of primary percutaneous coronary intervention (PCI), the mortality of ST‐elevation myocardial infarction (STEMI) has been greatly reduced [1]. Despite successful restoration of epicardial blood flow, some patients still exhibit inadequate myocardial tissue perfusion, a condition known as the no‐reflow phenomenon (NRP) [2]. The incidence rate of NRP among STEMI patients ranges from 10% to 30% [3]. Furthermore, NRP has been associated with more extensive myocardial injury, advanced heart failure (HF), and increased mortality [3, 4]. Therefore, there is an urgent need to find predictors of NRP in STEMI and to reduce the associated mortality. Beyond clinical risk scores, electrocardiographic parameters such as ST‐segment elevation in lead aVR may provide additional prognostic information in acute coronary syndrome (ACS) [5, 6]. However, simple, preprocedural clinical risk scores that integrate baseline comorbidity burden remain particularly valuable for early risk stratification and clinical decision‐making.

The CHA_2_DS_2_‐VASc score is clinically used in nonvalvular atrial fibrillation (AF) patients to assess stroke risk [7, 8, 9] and guide anticoagulation treatment [10]. In addition, studies further demonstrated its independent prognostic value for adverse events in both stable coronary artery disease (CAD) [11] and ACS [12], especially those with acute myocardial infarction [13, 14]. The 2024 European Society of Cardiology Guidelines for the Management of AF have updated the stroke risk stratification to the CHA_2_DS_2_‐VA score (hereafter referred to as the CV score) [15]. The CV score includes HF (1 point), hypertension (1 point), age ≥ 75 years (2 points), age > 65 years (1 point), diabetes mellitus (DM) (1 point), prior stroke (2 points), and vascular disease (1 point), excluding the former sex influence.

This study aimed to investigate the predictive ability of the preprocedural CV score for NRP in patients with STEMI undergoing primary PCI, in addition to subgroups according to sex differences.

## 2 Methods

### 2.1 Study Population

All consecutive patients with STEMI admitted to our hospital from 2015 July to August 2019 were screened for this retrospective study. Primary PCI was performed within 24 h following initial symptom presentation. STEMI was diagnosed in line with the ESC Guidelines [16]. Patients with hematological diseases, active infectious or inflammatory diseases, severe hepatic, renal, or respiratory dysfunction or failure, pregnancy, or active malignancy were excluded from this study. The clinical characteristics, including DM, hypertension, smoking, family history of ischemic heart disease (IHD), and laboratory characteristics, were obtained from the medical records database. This study obtained approval from the Ethics Committee of Liaocheng People's Hospital, and all procedures adhered to the ethical principles of the Declaration of Helsinki. All patients provided written informed consent.

### 2.2 Blood Sample Collection and Laboratory Testing

Blood samples were collected within 30 min of hospital admission and prior to primary PCI procedure. All laboratory parameters were obtained from peripheral venous blood samples using standard venipuncture techniques. The hospital's emergency laboratory then performed laboratory tests to measure a range of biochemical parameters, including d‐dimer, creatinine, blood glucose, total cholesterol (TC), and triglycerides (TG).

### 2.3 Angiographic Assessment of NRP During Primary PCI

During the coronary angiography procedure, two skilled invasive cardiologists, who were blinded to the CV scores and detailed clinical data, utilized the standard Judkins technique and iopamidol as the radiocontrast agent. The Thrombolysis in Myocardial Infarction (TIMI) flow score was employed to visually evaluate the scale of coronary blood flow. This grading system categorizes the flow score as follows: TIMI 0 indicates no antegrade blood flow, TIMI I suggesting possible blood flow, TIMI II signifies definite blood flow with incomplete perfusion, and TIMI III indicates complete perfusion [17, 18]. The definition of NRP was TIMI flow grade ≤ 2 with no vasospasm, stenosis, or dissection, or TIMI flow grade 3 with concomitant TIMI myocardial blush grade < 2 persisting post‐PCI [19, 20]. During this process, the number of lesion vessels, the number of stents implanted, the mean stent diameter, and the total length of the stents were calculated.

### 2.4 Statistical Analysis

Data were analyzed in SPSS 23.0 (IBM Corp., Armonk, NY, USA). The Shapiro−Wilk test evaluated the normality of continuous variables. Results are reported as mean ± standard deviation (normally distributed), median (interquartile range) (non‐normal), or frequency/percentage (categorical). Between‐group differences were analyzed using Chi‐squared (categorical) or Mann−Whitney *U* (continuous) tests. Predictors of NRP were first screened by univariate logistic regression; variables with *p* < 0.05 underwent multivariate modeling to derive adjusted odds ratios (AORs). A receiver operating characteristic (ROC) analysis was performed using SPSS software to further explore the applicability of the CV score as a potential biomarker for NRP. A complete‐case analysis was performed, and cases with missing data for key variables included in the multivariable model were excluded. So, no imputation method was used. The events‐per‐variable (EPV) rule was strictly followed during model construction. We adhered to the widely accepted criterion of a minimum of 10 events per variable. Given 85 events of NRP, the number of variables entered into the multivariable model was limited to a maximum of 8, and finally, six variables were included, satisfying the EPV requirement. To evaluate whether sex modified the association between the CV score and no‐reflow, a formal interaction term (sex × CV score) was tested. To assess the incremental predictive value of combining the CV score with other clinically relevant variables, we constructed a combined model and compared the area under the curve (AUC) values using the DeLong test. Statistical significance (*p* < 0.05) was assessed using two‐sided tests for all analyses.

## 3 Results

### 3.1 Patient Characteristics

**Figure 1:**
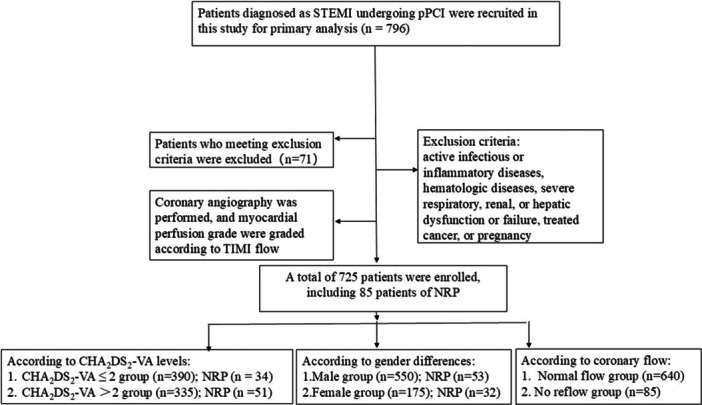
Flowchart of patient enrollment and study grouping.

Seven hundred and twenty‐five STEMI patients (550 males and 175 females) who underwent primary PCI were enrolled in the study (Figure 1). Two groups were defined by the median value of the CV score: ≤ 2 and > 2. Significant differences were observed in age, smoking status, DM, hypertension, CAD, sex, heart rate, hemoglobin, WBC counts, platelet, TC, d‐dimer, no‐reflow rate, number of lesion vessels, and mean stent diameter (Table 1). Additionally, we conducted a subgroup analysis based on sex, the differences in age, smoking status, DM, family history of IHD, hemoglobin, creatinine, TC levels, d‐dimer levels, no‐reflow rate, mean stent diameter, and CV score were significant, as shown in Table 2. Finally, we performed another subgroup analysis according to the coronary flow: the normal flow group and the NRP group. The results showed that age, family history of IHD, hemoglobin levels, platelet count, number of stents implanted, total length of stents, and the CV score were significantly different (Table 3).

**Table 1:** Basic characteristics of patients with CV differences.

Variable	≤ 2	> 2	*p* value
	*N* = 390	*N* = 335	
Age (year)	56 (14)	67 (13)	0.000*
Smoking, *n* (%)	237 (60.8)	161 (48.1)	0.001*
DM, *n* (%)	50 (12.8)	139 (41.5)	0.000*
Hypertension, *n* (%)	151 (38.7)	234 (69.9)	0.000*
CAD, *n* (%)	30(7.7)	52 (15.5)	0.001*
Family history of IHD, *n* (%)	34 (8.7)	17 (5.1)	0.059
Male, *n* (%)	315 (80.8)	235 (70.1)	0.001*
Heart rate (bpm)	78.17 ± 15.32	75 (18)	0.014*
Hemoglobin (g/L)	147 (19)	138.27 ± 17.27	0.000*
WBC count (×10^9^/L)	9.98 (3.99)	9.54 (3.98)	0.012*
NEU (×10^9^/L)	7.80 (4.00)	7.31 (4.05)	0.088
PLT (×10^9^/L)	236 (80)	218 (76)	0.004*
LYM (×10^9^/L)	1.38 (1.10)	1.33 (0.86)	0.363
Creatinine (umol/L)	61.80 (20)	64 (22)	0.057
TC (mmol/L)	4.77 (1.29)	4.62 (1.36)	0.018*
TG (mmol/L)	1.49 (1.26)	1.42 (1.20)	0.335
LVEF (%)	50 (10)	51 (9)	0.490
Fib (ng/mL)	2.95 (0.83)	3.04 (0.89)	0.121
d‐dimer (ng/mL)	0.30 (0.42)	0.37 (0.53)	0.011*
N/L	5.96 (6.16)	5.82 (5.15)	0.645
No‐reflow, *n* (%)	34 (8.7)	51 (15.2)	0.007*
Number of lesion vessels			
1, *n* (%)	129 (33.1)	97 (29)	0.048*
2, *n* (%)	148 (37.9)	112 (33.4)	
3, *n* (%)	113 (29)	126 (37.6)	
Number of stents implanted			
1, *n* (%)	314 (80.5)	261 (77.9)	0.658
2, *n* (%)	71 (18.2)	70 (20.9)	
3, *n* (%)	5 (1.3)	4 (1.27)	
Mean stent diameter, mm	3 (0.75)	3 (0.35)	0.015*
Total length of stents, mm	28 (16)	30 (18)	0.335
Time‐to PCI, h	4 (4)	4 (4)	0.257

*Note:* Normally distributed numerical variables were expressed as mean ± standard deviation, while non‐normally distributed data were expressed as median (interquartile range). Categorical variables were reported in frequency (percentages) and also marked as *n* (%) in the first column.

Abbreviations: CAD, coronary artery disease; DM, diabetes mellitus; Fib, fibrinogen; IHD, ischemic heart disease; LVEF, left ventricular ejection fraction; LYM, lymphocyte; NEU, neutrophils; N/L, neutrophils to lymphocyte ratio; PCI, percutaneous coronary intervention; PLT, platelet; TC, total cholesterol; TG, triglyceride; WBC, white blood cell.

* *p*<0.05.

**Table 2:** Basic characteristics of patients with sex differences.

Variable	Male	Female	*p* value
	*N* = 550	*N* = 175	
Age (year)	59 (17)	66.97 ± 8.17	< 0.001*
Smoking, *n* (%)	387 (70.4)	11 (6.3)	< 0.001*
DM, *n* (%)	133 (24.2)	56 (32.0)	0.040*
Hypertension, *n* (%)	286 (52)	99 (56.6)	0.291
CAD, *n* (%)	58 (10.5)	24 (13.7)	0.249
Family history of IHD, *n* (%)	48 (8.7)	3 (1.7)	0.003*
Heart rate (bpm)	76 (21)	73.85 ± 15.79	0.396
Hemoglobin (g/L)	147 (18)	131 (19)	< 0.001*
WBC count (×10^9^/L)	9.96 (4.13)	9.71 ± 2.81	0.082
NEU (×10^9^/L)	7.53 (4.14)	7.70 ± 2.80	0.492
PLT (×10^9^/L)	223 (77)	238 (89)	0.055
LYM (×10^9^/L)	1.38 (1.00)	1.26 (0.87)	0.248
Creatinine (umol/L)	66.0 (19)	53.4 (17)	< 0.001*
TC (mmol/L)	4.63 (1.20)	5.09 ± 1.03	< 0.001*
TG (mmol/L)	1.47 (1.27)	1.43 (1.15)	0.918
LVEF (%)	50 (9)	50 (10)	0.332
Fib (ng/mL)	2.98 (0.87)	3.04 (0.82)	0.124
d‐dimer (ng/mL)	0.30 (0.43)	0.41(0.56)	< 0.001*
N/L	6.00 (5.49)	5.78 (5.64)	0.868
No‐reflow, *n* (%)	53 (9.6)	32(18.3)	0.002*
Number of lesion vessels			
1, *n* (%)	176 (32)	50 (28.6)	0.228
2, *n* (%)	202 (36.7)	58 (33.1)	
3, *n* (%)	172 (31.3)	67 (38.3)	
Number of stents implanted			
1, *n* (%)	438 (79.6)	137 (78.3)	0.547
2, *n* (%)	104 (18.9)	37 (21.1)	
3, *n* (%)	8 (1.5)	1 (0.6)	
Mean stent diameter, mm	3 (0.75)	3 (0.25)	0.012*
Total length of stents, mm	28.5 (16)	30 (16)	0.726
Time‐to PCI, h	3.5 (4)	4 (3.5)	0.163
CHA_2_DS_2_‐VA Score	2 (1)	3 (2)	< 0.001*

*Note:* Normally distributed numerical variables were expressed as mean ± standard deviation, while non‐normally distributed data were expressed as median (interquartile range). Categorical variables were reported in frequency (percentages) and also marked as *n* (%) in the first column.

Abbreviations: CAD, coronary artery disease; DM, diabetes mellitus; Fib, fibrinogen; IHD, ischemic heart disease; LVEF, left ventricular ejection fraction; LYM, lymphocyte; NEU, neutrophils; N/L, neutrophils to lymphocyte ratio; PCI, percutaneous coronary intervention; PLT, platelet; TC, total cholesterol; TG, triglyceride; WBC, white blood cell.

*p < 0.05.

**Table 3:** Basic characteristics of patients with TIMI difference.

Variable	Normal‐ flow	No‐reflow	*p* value
	*N* = 640	*N* = 85	
Age (year)	61 (17)	65.20 ± 9.83	< 0.001*
DM, *n* (%)	165 (25.8)	24 (28.2)	0.628
Hypertension, *n* (%)	337 (52.7)	48 (56.5)	0.508
CAD, *n* (%)	7 (11.1)	11 (12.9)	0.613
Smoking, *n* (%)	356 (55.6)	42 (49.4)	0.279
Family history of IHD, *n* (%)	51 (8)	0 (0)	0.007*
Heart rate (bpm)	76 (20)	74.82 ± 17.28	0.149
Hemoglobin (g/L)	144 (21)	136.44 ± 16.00	< 0.001*
WBC count (×10^9^/L)	9.90 (3.88)	9.66 ± 3.15	0.218
NEU (×10^9^/L)	7.54 (3.93)	7.71 ± 3.07	0.683
PLT (×10^9^/L)	231 (78)	202 (75)	0.002*
LYM (×10^9^/L)	1.36 (0.98)	1.3 (0.94)	0.063
Creatinine (umol/L)	62.6 (20)	65 (20)	0.407
TC (mmol/L)	4.72 (1.34)	4.74 ± 0.86	0.776
TG (mmol/L)	1.46 (1.24)	1.48 (1.24)	0.677
LVEF (%)	50 (9)	50 (10)	0.506
Fib (ng/mL)	2.98 (0.84)	3.09 (1.00)	0.076
d‐dimer (ng/mL)	0.314 (0.41)	0.40(0.61)	0.293
N/L	5.75 (5.61)	6.42 (583)	0.328
Number of lesion vessels			
1, *n* (%)	200 (31.3)	26 (30.6)	0.491
2, *n* (%)	225 (35.2)	35 (41.2)	
3, *n* (%)	215 (33.5)	24 (28.2)	
Number of stents implanted			
1, *n* (%)	517 (80.8)	58 (68.2)	0.006*
2, *n* (%)	114 (17.8)	27 (31.8)	
3, *n* (%)	9 (1.4)	0 (0)	
Mean stent diameter, mm	3 (0.5)	3 (0.63)	0.766
Total length of stents, mm	28 (16)	33.44 ± 12.80	0.017*
Time‐to PCI, h	4 (4)	4 (3.25)	0.206
CHA_2_DS_2_‐VA Score	2 (1)	3 (2)	< 0.001*
Cardiogenic shock, *n* (%)	6 (0.9)	2 (2.4)	0.535

*Note:* Normally distributed numerical variables were expressed as mean ± standard deviation, while non‐normally distributed data were expressed as median (interquartile range). Categorical variables were reported in frequency (percentages) and also marked as *n* (%) in the first column.

Abbreviations: CAD, coronary artery disease; DM, diabetes mellitus; Fib, fibrinogen; IHD, ischemic heart disease; LVEF, left ventricular ejection fraction; LYM, lymphocyte; NEU, neutrophils; N/L, neutrophils to lymphocyte ratio; PCI, percutaneous coronary intervention; PLT, platelet; TC, total cholesterol; TG, triglyceride; WBC, white blood cell.

*p < 0.05.

### 3.2 Rate of NRP Among the Studied Subjects

The incidence of NRP was calculated to be 11.7% (85 out of 725 patients) for all patients in the study. In subgroup analysis, the incidence of NRP was 8.7% (34/390) in the low CV score group versus 15.2% (51/335) in the high CV score group (*p* = 0.007) (Table 1). Additionally, sex differences were examined, revealing an incidence of 53 in males and 32 in females. This difference was also statistically significant (9.6% vs. 18.3%, *p* = 0.002) (Table 2). A significant difference was also found in the CV score between normal flow and the NRP group (*p* < 0.001), as shown in Table 3.

### 3.3 Analysis to Illustrate Predictors of the NRP

In all studied subjects, the univariate logistic regression analysis indicated that the CV score was a predictor for NRP (OR: 1.365, 95% confidence interval [CI]: 1.167–1.598, *p* < 0.001). Additionally, sex (OR: 2.098, 95% CI: 1.303–3.380, *p* = 0.002), hemoglobin (OR: 0.979, 95% CI: 0.967–0.992, *p* = 0.001), platelets (OR: 0.995, 95% CI: 0.991–0.999, *p* = 0.012), lymphocyte (OR: 0.746, 95% CI: 0.556–0.999, *p* = 0.049), and number of stents implanted (OR: 1.641, 95% CI: 1.046–2.576, *p* = 0.031) were predictors of NRP. Multivariate logistic regression analysis model including the CV score, sex, hemoglobin, platelets, lymphocytes, and number of stents implanted showed that the CV score (AOR: 1.295, 95% CI: 1.098–1.527, *p* = 0.002), sex (AOR: 1.831, 95% CI: 1.061–3.158, *p* = 0.030), platelets (AOR: 0.995, 95% CI: 0.991–1.000, *p* = 0.031), and number of stents implanted (AOR: 1.694, 95% CI: 1.061−2.705, *p* = 0.027) were independent predictors of NRP (Table 4). The variance inflation factor values for all the variables included in the model were less than 2.0, indicating no significant multicollinearity. Both continuous and categorical modeling confirmed a proportional, dose−response relationship between the CV score and NRP risk, reinforcing the robustness of our findings.

**Table 4:** Logistic regression analysis of predictors of no‐reflow in the overall studied subjects.

Variables	OR	95% CI	*p* value	Adjusted OR	95% CI	*p* value
CHA_2_DS_2_‐VA Score	1.365	1.167–1.598	< 0.001	1.295	1.098–1.527	0.002*
Sex	2.098	1.303–3.38	0.002	1.831	1.061–3.158	0.030*
Smoking	0.779	0.495–1.226	0.280			
Heart rate	0.990	0.976–1.005	0.184			
WBC	0.946	0.875–1.022	0.156			
Hemoglobin	0.979	0.967–0.992	0.001	0.992	0.977–1.007	0.278
PLT	0.995	0.991–0.999	0.012	0.995	0.991–1.000	0.031*
NEU	0.973	0.903–1.049	0.482			
LYM	0.746	0.556–0.999	0.049	0.832	0.610–1.135	0.246
TC	0.934	0.751–1.162	0.541			
TG	0.974	0.824–1.151	0.756			
Cr	1.006	0.994–1.019	0.320			
Fib	1.240	0.954–1.611	0.107			
d‐dimer	1.172	0.971–1.414	0.098			
N/L	1.019	0.973–1.067	0.427			
LVEF	0.999	0.969–1.029	0.931			
Mean stent diameter	1.194	0.679–2.100	0.538			
Total length of stents	1.014	0.999–1.029	0.077			
Number of stents implanted	1.641	1.046–2.576	0.031	1.694	1.061–2.705	0.027*
Number of lesion vessels	0.929	0.701–1.233	0.611			
Infarct location	1.256	0.797–1.977	0.326			
Killip class > 2	1.660	0.667–4.134	0.276			
Time‐to PCI	1.019	0.967–1.073	0.490			

Abbreviations: CAD, coronary artery disease; CI, confidence interval; Cr, creatinine; DM, diabetes mellitus; Fib, fibrinogen; Infarct location, 1 anterior wall; 0 non‐anterior wall; LVEF, left ventricular ejection fraction; LYM, lymphocyte; NEU, neutrophils; N/L, neutrophils to lymphocyte ratio; OR, odds ratio; PCI, percutaneous coronary intervention; PLT, platelet; TC, total cholesterol; TG, triglyceride; WBC, white blood cell.

*p < 0.05

Internal validation using 1000 bootstrap resamples confirmed the stability of the multivariable logistic regression model. The bootstrap‐derived OR for the CV score was 1.294 (95% BCa CI: 1.071–1.531, *p* = 0.002), which was highly consistent with the original adjusted OR of 1.295 (95% CI: 1.098–1.527, *p* = 0.002). Similarly, other significant predictors showed robust and consistent results between bootstrap and primary analyses. These findings support the reliability and internal validity of the predictive model.

Regarding the subgroup analysis based on sex, in the female group, the CV score (AOR: 1.597, 95% CI: 1.187–2.149, *p* = 0.002) and creatinine (AOR: 1.037, 95% CI: 1.009–1.066, *p* = 0.009) were independent predictors of NRP (Table 5). In contrast, among male patients, only platelet count (AOR: 0.993, 95% CI: 0.987–0.999, *p* = 0.033), but not the CV score, was an independent predictor of NRP (Table 6).

**Table 5:** Logistic regression analysis of predictors of no‐reflow in the female population.

Variables	OR	95% CI	*p* value	Adjusted OR	95% CI	*p* value
CHA_2_DS_2_‐VA Score	1.620	1.217–2.155	0.001	1.597	1.187–2.149	0.002*
Smoking	0.993	0.204–4.831	0.993			
DM	0.958	0.420–2.189	0.920			
Hypertension	1.866	0.833–4.267	0.128			
CAD	0.624	0.226–1.723	0.363			
Heart rate	0.989	0.965–1.014	0.385			
WBC count	1.121	0.983–1.279	0.089			
Hemoglobin	1.001	0.976–1.027	0.920			
PLT	1.0	0.995–1.005	0.938			
NEU	1.117	0.975–1.279	0.110			
LYM	1.006	0.660–1.533	0.978			
TC	0.984	0.677–1.431	0.932			
TG	1.162	0.854–1.582	0.340			
Cr	1.039	1.012–1.066	0.004	1.037	1.009–1.066	0.009*
Fib	1.054	0.663–1.677	0.823			
d‐dimer	1.194	0.931–1.531	0.162			
N/L	1.020	0.934–1.114	0.659			
LVEF	0.974	0.929–1.022	0.289			
Mean stent diameter	2.35	0.879–6.283	0.089			
Total length of stents	1.013	0.986–1.041	0.343			
Number of stents implanted	1.190	0.502–2.818	0.693			
Number of lesion vessels	1.013	0.986–1.041	0.343			
Infarct location	1.523	0.706–3.288	0.283			
Killip class > 2	1.560	0.468–5.193	0.469			
Time‐to PCI	0.938	0.815–1.079	0.371			

Abbreviations: CAD, coronary artery disease; CI, confidence interval; Cr, creatinine; DM, diabetes mellitus; Fib, fibrinogen; Infarct location, 1 anterior wall; 0 non‐anterior wall; LVEF, left ventricular ejection fraction; LYM, lymphocyte; NEU, neutrophils; N/L, neutrophils to lymphocyte ratio; OR, odds ratio; PCI, percutaneous coronary intervention; PLT, platelet; TC, total cholesterol; TG, triglyceride; WBC, white blood cell.

*p < 0.05.

**Table 6:** Logistic regression analysis of predictors of no‐reflow in the male population.

Variables	OR	95% CI	*p* value	Adjusted OR	95% CI	*p* value
CHA_2_DS_2_‐VA Score	1.219	0.999–1.488	0.052			
Smoking	1.330	0.691–2.559	0.393			
DM	1.140	0.599–2.172	0.690			
Hypertension	0.878	0.498–1.575	0.652			
CAD	0.873	0.333–2.228	0.782			
Heart rate	0.992	0.974–1.010	0.384			
WBC	0.873	0.786–0.968	0.010	0.964	0.860–1.080	0.524
Hemoglobin	0.977	0.960–0.994	0.008	0.990	0.970–1.010	0.318
PLT	0.990	0.984–0.995	0.000	0.993	0.987–0.999	0.033*
NEU	0.916	0.830–1.011	0.081			
LYM	0.620	0.412–0.933	0.022	0.790	0.524–1.191	0.260
TC	0.825	0.617–1.104	0.195			
TG	0.917	0.737–1.140	0.434			
Cr	1.005	0.990–1.021	0.498			
Fib	1.318	0.960–1.809	0.088			
d‐dimer	1.033	0.736–1.450	0.851			
N/L	1.021	0.965–1.079	0.472			
LVEF	1.017	0.978–1.057	0.400			
Mean stent diameter	0.976	0.474–2.013	0.948			
Total length of stents	1.015	0.996–1.034	0.117			
Number of stents implanted	1.899	1.114–3.238	0.018	1.844	1.055–3.223	0.320
Number of lesion vessels	1.015	0.996–1.034	0.117			
Infarct location	1.203	0.681–2.125	0.523			
Killip class > 2	1.179	0.264–5.273	0.829			
Time‐to PCI	1.040	0.982–1.100	0.180			

Abbreviations: CAD, coronary artery disease; CI, confidence interval; Cr, creatinine; DM, diabetes mellitus; Fib, fibrinogen; Infarct location, 1 anterior wall; 0 non‐anterior wall; LVEF, left ventricular ejection fraction; LYM, lymphocyte; NEU, neutrophils; N/L, neutrophils to lymphocyte ratio; OR, odds ratio; PCI, percutaneous coronary.

*p < 0.05.

A formal interaction term for sex × CV score was tested in the multivariable model including CV score, sex, their interaction term, and clinically relevant covariates (infarct location, hemoglobin, platelet count, lymphocyte count, and number of stents implanted). Results showed that the interaction was statistically significant (OR = 1.569, 95% CI: 1.082–2.275, *p* for interaction = 0.017). This result indicated that the association between the CV score and no‐reflow is significantly stronger in females than in males.

### 3.4 ROC Curve for the CV Score to Determine NRP

**Figure 2:**
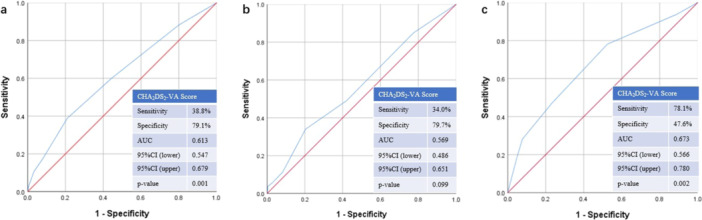
Receiver operating characteristic (ROC) curves of CHA_2_DS_2_‐VA score for predicting no‐reflow phenomenon (NRP) in different subgroups: (a) ROC for CV score to determine NRP in the over cohort, (b) ROC for CV score to determine NRP in the male group,(c) ROC for CV score to determine NRP in the female group.

In the overall cohort, the AUC of CV score to predict NRP was 0.613, with 38.8% sensitivity, 79.1% specificity (*p* = 0.001) (Figure 2a). In the subgroup analysis, the AUC in male patients was 0.569 (*p* = 0.099), indicating no significant discriminative ability (Figure 2b). However, in the female group, the AUC of the CV score predicting NRP was 0.673 (78.1% sensitivity and 47.6% specificity (*p* = 0.002) (Figure 2c).

To evaluate whether the integration of complementary markers improves predictive performance, we compared the CV score alone (Model 1) with a combined model incorporating sex, hemoglobin, platelet count, lymphocyte count, and number of stents implanted (Model 2) (Table S1 and Figure S1). In the overall cohort, Model 2 achieved a significantly higher AUC than Model 1 (0.689 vs. 0.613, DeLong *p* = 0.005), indicating improved discriminative ability with the addition of laboratory and procedural variables. However, subgroup analysis revealed distinct patterns by sex. In female patients, the CV score alone demonstrated a discriminative ability (AUC = 0.673, *p* = 0.002), and the addition of complementary markers did not significantly improve prediction (AUC = 0.682, DeLong *p* = 0.500). In contrast, among male patients, the CV score alone showed poor discriminative ability (AUC = 0.569, *p* = 0.099), whereas Model 2 achieved substantially better performance (AUC = 0.721, DeLong *p* = 0.001). Shown in Table S1 and Figure S1. These findings suggest that the incremental value of a combined model is predominantly observed in male patients.

## 4 Discussion

Our study demonstrates that the CV score is an independent predictor of no‐reflow in female STEMI patients undergoing primary PCI, but not in males. The significant sex × CV score interaction indicates that the CV score operates as a sex‐modified predictor, with its prognostic information concentrated in the female subgroup, where accumulated comorbidities translate into higher microvascular risk. This sex‐specific pattern is further corroborated by the DeLong test comparison: in females, the CV score alone achieved adequate discrimination without incremental gain from added markers, whereas in males, standalone discrimination was poor, but improved substantially with a combined model (AUC > 0.7). These findings suggest that no‐reflow in males is driven less by baseline comorbidity burden than by acute procedural and hematological factors.

The NRP is a critical determinant of prognosis in AMI [4, 21]. Its established risk factors: DM [22], older age [23, 24], hypertension [24], and vascular disease [12] are incorporated into the CHA_2_DS_2_‐VASc score, which has shown predictive value for NRP in prior studies [25], though findings remain inconsistent [26, 27, 28, 29]. The 2024 ESC AF guidelines [15] removed the female sex category from this score, yielding the CV score. In the present study, the CV score predicted NRP in the overall cohort, but this effect was confined to females. The significant sex × CV score interaction indicates that the association between comorbidity burden and NRP is stronger in females, where accumulated cardiovascular risk translates into higher microvascular vulnerability [3, 30, 31, 32, 33, 34]. In males, NRP appears driven more by acute procedural factors than by baseline comorbidities [30, 31, 35]. These findings support the use of the CV score in female STEMI patients as a standalone risk stratification tool, while suggesting that male patients require additional laboratory and procedural markers for adequate prediction.

Sex‐related differences have long been recognized as a crucial factor influencing of STEMI outcomes [35]. Research has consistently demonstrated that female sex is an independent predictor of worse short‐term and long‐term outcomes following AMI, including higher 30‐day mortality and less favorable baseline characteristics [36, 37]. One study investigated the NRP group after primary PCI and found that females with TIMI grade 1 had a higher incidence of impaired ejection fraction, AF, and tachycardia than males [38]. In addition, the study also demonstrated that the TIMI grade 1 group had a higher incidence of in‐hospital mortality in females than in males. In our cohort, the female group had a higher incidence of NRP compared with males (18.3% vs. 9.6%), consistent with prior studies [37].

The significant sex × CHA_2_DS_2_‐VA interaction provides robust statistical evidence that the predictive effect of the CV score is quantitatively and qualitatively different between women and men. Specifically, each one‐point increment in the CV score conferred a 56.9% greater odds of no‐reflow in females compared with males after adjustment for infarct location, hemoglobin, platelet count, lymphocyte count, and stent number. This interaction is clinically meaningful because it indicates that the CV score operates as a sex‐modified predictor: its prognostic information is concentrated in the female subgroup, where accumulated cardiovascular comorbidities (hypertension, diabetes, HF, vascular disease) translate into a higher intrinsic risk of microvascular dysfunction. In males, the absence of a significant association suggests that baseline comorbidity burden, as captured by the CV score, is not the dominant driver of no‐reflow; rather, acute procedural and hematological factors appear to predominate. These findings align with contemporary evidence that no‐reflow pathophysiology is sex‐divergent [38], with women exhibiting greater microvascular vulnerability to chronic risk factors and men exhibiting greater susceptibility to acute thromboembolic and procedural insults.

The predictive performance of the CV score alone was modest in the overall cohort, consistent with the inherent limitations of any single baseline clinical score. However, our model comparison analysis revealed important sex‐specific differences in the incremental value of combining the CV score with complementary markers. In female patients, the CV score alone provided a discriminative ability, and the addition of laboratory and procedural variables did not significantly improve prediction (DeLong *p* = 0.500). This suggests that the CV score captures the majority of predictive information in women, likely because female patients in our cohort presented at an older age with a greater accumulation of cardiovascular comorbidities, making the comorbidity‐based score particularly informative. In contrast, male patients showed poor discriminative ability with the CV score alone, but achieved substantially improved prediction when laboratory and procedural factors were integrated. This sex discrepancy likely reflects differences in the underlying pathophysiology of no‐reflow. In men, acute procedural factors, such as distal atherothrombotic embolization and thrombus burden [39, 40], may play a more prominent role than baseline comorbidity burden. Consequently, male patients appear to benefit most from a comprehensive combined model that incorporates both clinical risk stratification and acute procedural/laboratory markers.

The mechanisms underlying the female‐specific predictive value of the CV score for no‐reflow are multifactorial. First, women are characterized by a higher prevalence of coronary microvascular dysfunction, which is closely related to estrogen deficiency, smaller coronary vascular dimensions, and increased microvascular resistance [41]. Recent expert panel statements and mechanistic reviews confirm that loss of estrogen‐dependent endothelial protection disproportionately raises microvascular resistance in women, making the coronary microcirculation more vulnerable to the comorbidities captured by the CV score, such as hypertension, diabetes, HF, and vascular disease [41]. Second, females exhibit stronger platelet reactivity, higher thrombotic burden, and more frequent distal atherothrombotic embolization during primary PCI [35, 42]. Large‐scale registry data show that thrombus burden remains one of the strongest procedural predictors of no‐reflow, and its interaction with baseline platelet hyper‐reactivity may be particularly marked in women [35]. These factors further exacerbate microvascular obstruction when the comorbidity burden is already high. Third, women with STEMI more often present with diffuse atherosclerosis, plaque erosion, and systemic inflammation rather than typical plaque rupture [43, 44, 45, 46]. Multimodality imaging studies have demonstrated that perivascular inflammation is significantly associated with thin‐cap fibroatheroma and macrophage accumulation in women, whereas this link is not observed in men [43]. Collectively, these features create a pathophysiological substrate that amplifies ischemia/reperfusion injury and no‐reflow [47]. Taken together, these factors account for why the CV score independently predicts no‐reflow in females but not in males.

These findings have practical implications for risk stratification in daily clinical practice. For female STEMI patients, the preprocedural CV score may serve as a simple and effective standalone screening tool to identify high‐risk individuals. For male patients, however, clinicians should consider integrating the CV score with readily available laboratory parameters and procedural characteristics to achieve acceptable predictive accuracy. Future prospective studies are warranted to validate this sex‐specific approach and to determine whether a combined multi‐modality risk model can improve clinical decision‐making and patient outcomes.

### 4.1 Study Limitations

This study had several limitations. First, this was a retrospective, single‐center observational study with a relatively limited sample size, which may introduce selection bias and restrict the generalizability of the conclusions. Second, the present study lacked external validation in an independent patient cohort; therefore, future large‐scale, multicenter, prospective studies are needed to verify the stability and applicability of the CV score for predicting NRP. Third, the predictive performance represented by AUC values was 0.613 in the overall population and 0.673 in females, suggesting weak‐to‐moderate discriminative ability, even though the results were statistically significant. Fourth, the diagnosis of no‐reflow was exclusively based on TIMI flow grade and myocardial blush grade, whereas ST‐segment resolution and other microvascular perfusion markers were not assessed. Fifth, although multivariable regression was used to adjust for potential confounders, residual or unmeasured confounding factors cannot be completely excluded. Finally, the precise pathophysiological mechanisms linking the CV score to no‐reflow in females remain incompletely understood and require further basic and clinical research. Thus, more research is needed to further elucidate the mechanisms between the CV score and NRP in STEMI.

## 5 Conclusion

The present study demonstrates that the CV score is an independent predictor of NRP in female STEMI patients undergoing primary PCI, but not in males. In females, the CV score alone provides adequate risk stratification, whereas in males, integration with laboratory and procedural markers is required to achieve acceptable predictive accuracy. These sex‐specific findings support a tailored approach to no‐reflow risk assessment in clinical practice.

## Author Contributions

Ying Sun was responsible for the study design, drafted the manuscript, and conducted the statistical analysis. Wei Wang, Jian Ren, and Chunsong Wang interpreted the data. Min Liu and Jiahui Liu analyzed the findings and drafted the manuscript. Hengchen Yao designed, and Xuewen Qi supervised the study. All authors gave final approval for this version to be published. Hengchen Yao and Xuewen Qi are the guarantors of this work, responsible for the data of the study.

## Conflicts of Interest

The authors declare no conflicts of interest.

## Supporting information


**Figure S1:** Receiver operating characteristic (ROC) curves comparing the predictive performance of the CHA_2_DS_2_‐VA (CV) score alone (Model 1) and the full combined model (Model 2) for no‐reflow phenomenon in ST‐elevation myocardial infarction patients undergoing primary percutaneous coronary intervention. Model 1 = CV score alone; Model 2 = CV score combined with sex, hemoglobin, platelet count, lymphocyte count, and number of stents implanted. AUC = area under the curve

Supporting File

## Data Availability

The data supporting this study are available from the corresponding author upon reasonable request.

## References

[1] P. G. Thrane , K. K. W. Olesen , T. Thim , et al., “Mortality Trends After Primary Percutaneous Coronary Intervention for ST‐Segment Elevation Myocardial Infarction,” Journal of the American College of Cardiology 82 (2023): 999–1010. 37648359 10.1016/j.jacc.2023.06.025

[2] G. Annibali , I. Scrocca , T. C. Aranzulla , E. Meliga , F. Maiellaro , and G. Musumeci , “‘No‐Reflow’ Phenomenon: A Contemporary Review,” Journal of Clinical Medicine 11 (2022): 2233. 35456326 10.3390/jcm11082233 PMC9028464

[3] G. Ndrepepa and A. Kastrati , “Coronary No‐Reflow After Primary Percutaneous Coronary Intervention: Current Knowledge on Pathophysiology, Diagnosis, Clinical Impact and Therapy,” Journal of Clinical Medicine 12 (2023): 5592. 37685660 10.3390/jcm12175592 PMC10488607

[4] L. R. Pantea‐Roșan , V. A. Pantea , S. Bungau , et al., “No‐Reflow After PPCI‐A Predictor of Short‐Term Outcomes in STEMI Patients,” Journal of Clinical Medicine 9 (2020): 2956. 32932736 10.3390/jcm9092956 PMC7563881

[5] A. Wang , V. Singh , Y. Duan , et al., “Prognostic Implications of ST‐Segment Elevation in Lead aVR in Patients With Acute Coronary Syndrome: A Meta‐Analysis,” Annals of Noninvasive Electrocardiology 26 (2021): e12811. 33058358 10.1111/anec.12811 PMC7816815

[6] Y. K. İçen , Y. Dönmez , A. O. Demirtaş , et al., “Ischemic Changes in Lead aVR Is Associated With Left Ventricular Thrombus or High‐Grade Spontaneous Echocontrast in Patients With Acute Anterior Myocardial Infarction,” Türk Kardiyoloji Derneği Arşivi: Türk Kardiyoloji Derneğinin Yayın Organıdır 2019, no. 47: 168–176. 10.5543/tkda.2018.57296 30982814

[7] K. Teppo , G. Y. H. Lip , K. E. J. Airaksinen , et al., “Comparing CHA_2_DS_2_‐VA and CHA_2_DS_2_‐VASc Scores for Stroke Risk Stratification in Patients With Atrial Fibrillation: A Temporal Trends Analysis,” Lancet Regional Health ‐ Europe 43 (2024): 100967. 39171253 10.1016/j.lanepe.2024.100967 PMC11337097

[8] W. H. Cheng , Y. H. Chan , L. Kuo , et al., “Stroke Risks in Women vs Men in Asian Patients With Atrial Fibrillation: A Temporal Trend Analysis and a Comparison of the CHA2DS2‐VASc and CHA2DS2‐VA Stroke Risk Stratification Scores,” Heart Rhythm: The Official Journal of the Heart Rhythm Society 22 (2025): 2515–2523. 10.1016/j.hrthm.2025.05.067 40466843

[9] G. Y. H. Lip , R. Nieuwlaat , R. Pisters , D. A. Lane , and H. J. G. M. Crijns , “Refining Clinical Risk Stratification for Predicting Stroke and Thromboembolism in Atrial Fibrillation Using a Novel Risk Factor ‐ Based Approach,” Chest 137 (2010): 263–272. 19762550 10.1378/chest.09-1584

[10] H. Tomita and K. Okumura , “Current Risk Stratification Schemes for Preventing Ischemic Stroke in Patients with Nonvalvular Atrial Fibrillation: HELT ‐ E_2_S_2_ and CHA2DS2 ‐ VA Scores,” Journal of Cardiology 87 (2026): 136–143. 41138935 10.1016/j.jjcc.2025.10.005

[11] J. J. Sanchez Fernandez , M. R. Ortiz , F. M. Ballesteros , et al., “CHA(2)DS(2) ‐ VASc Score as Predictor of Stroke and All ‐ Cause Death in Stable Ischaemic Heart Disease Patients Without Atrial Fibrillation,” Journal of Neurology 267 (2020): 3061–3068. 32529579 10.1007/s00415-020-09961-7

[12] X. Huang , W. Zheng , X. D. Zhao , and S. P. Nie , “CHA2DS2 ‐ VASc Score Predicts the Slow Flow/No ‐ Reflow Phenomenon in ST ‐ Segment Elevation Myocardial Infarction Patients with Multivessel Disease Undergoing Primary Percutaneous Coronary Intervention,” Medicine 100 (2021): e26162. 34032776 10.1097/MD.0000000000026162 PMC8154372

[13] C. Fang , Z. Chen , J. Zhang , X. Jin , and M. Yang , “Association of CHA2DS2 ‐ VASC Score with In ‐ Hospital Cardiovascular Adverse Events in Patients with Acute ST ‐ Segment Elevation Myocardial Infarction,” International Journal of Clinical Practice 2022 (2022): 3659381. 36225534 10.1155/2022/3659381 PMC9525758

[14] M. K. Akboga , S. Yilmaz , and R. Yalcin , “Prognostic Value of CHA2DS2 ‐ VASc Score in Predicting High SYNTAX Score and In ‐ Hospital Mortality for Non ‐ ST Elevation Myocardial Infarction in Patients Without Atrial Fibrillation,” Anatolian Journal of Cardiology 25 (2021): 789–795. 34734812 10.5152/AnatolJCardiol.2021.03982 PMC8575397

[15] I. C. Van Gelder , M. Rienstra , K. V. Bunting , et al., “ESC Guidelines for the Management of Atrial Fibrillation Developed in Collaboration with the European Association for Cardio ‐ Thoracic Surgery (EACTS),” European Heart Journal 45, no. 2024 (2024): 3314–3414. 39210723 10.1093/eurheartj/ehae176

[16] B. Ibanez , S. James , S. Agewall , et al., “ESC Guidelines for the Management of Acute Myocardial Infarction in Patients Presenting with ST ‐ Segment Elevation: The Task Force for the Management of Acute Myocardial Infarction in Patients Presenting with ST ‐ Segment Elevation of the European Society of Cardiology (ESC),” European Heart Journal 39, no. 2018 (2017): 119–177. 10.1093/eurheartj/ehx393 28886621

[17] C. M. Gibson , C. P. Cannon , W. L. Daley , et al., “TIMI Frame Count: A Quantitative Method of Assessing Coronary Artery Flow,” Circulation 93 (1996): 879–888. 8598078 10.1161/01.cir.93.5.879

[18] G.TIMI Study ., “The Thrombolysis in Myocardial Infarction (TIMI) Trial. Phase I Findings,” New England Journal of Medicine 312 (1985): 932–936. 4038784 10.1056/NEJM198504043121437

[19] H. R. Hellstrom , “No ‐ Reflow Phenomenon,” Circulation 106 (2002): e143. 12427665 10.1161/01.cir.0000037128.03240.41

[20] H. A. W. van't, A. Liem , H. Suryapranata , J. C. Hoorntje , M. J. de Boer , and F. Zijlstra , “Angiographic Assessment of Myocardial Reperfusion in Patients Treated with Primary Angioplasty for Acute Myocardial Infarction: Myocardial Blush Grade, Zwolle Myocardial Infarction Study Group,” Circulation 97 (1998): 2302–2306. 9639373 10.1161/01.cir.97.23.2302

[21] H. Liu , F. Zhang , Y. Li , et al., “The HALP Score Predicts No ‐ Reflow Phenomenon and Long ‐ Term Prognosis in Patients with ST ‐ Segment Elevation Myocardial Infarction After Primary Percutaneous Coronary Intervention,” Coronary Artery Disease 36 (2025): 273–280. 39492724 10.1097/MCA.0000000000001446 PMC12043261

[22] A. Dziewierz , B. Zdzierak , K. P. Malinowski , et al., “Diabetes Mellitus Is Still a Strong Predictor of Periprocedural Outcomes of Primary Percutaneous Coronary Interventions in Patients Presenting with ST ‐ Segment Elevation Myocardial Infarction (From the ORPKI Polish National Registry),” Journal of Clinical Medicine 11 (2022): 6284. 36362512 10.3390/jcm11216284 PMC9657628

[23] P. Aggarwal , L. Rekwal , S. K. Sinha , R. K. Nath , D. Khanra , and A. P. Singh , “Predictors of No ‐ Reflow Phenomenon Following Percutaneous Coronary Intervention for ST ‐ Segment Elevation Myocardial Infarction,” Annales de Cardiologie et d'Angéiologie 70 (2021): 136–142. 10.1016/j.ancard.2021.04.004 33962782

[24] J. A. Rossington , E. Sol , K. Masoura , et al., “No ‐ Reflow Phenomenon and Comparison to the Normal ‐ Flow Population Postprimary Percutaneous Coronary Intervention for ST Elevation Myocardial Infarction: Case ‐ Control Study (NORM PPCI),” Open Heart 7 (2020): e001215. 32719072 10.1136/openhrt-2019-001215 PMC7380712

[25] Q. Zhang , M. Hu , S. Ma , and T. Niu , “New R(2) ‐ CHA(2)DS(2) ‐ VASc Score Predicts No ‐ Reflow Phenomenon and Long ‐ Term Prognosis in Patients With ST ‐ Segment Elevation Myocardial Infarction After Primary Percutaneous Coronary Intervention,” Frontiers in Cardiovascular Medicine 9, no. 0 (2022): 899739. 36312233 10.3389/fcvm.2022.899739 PMC9609412

[26] M. I. Rashed , M. A. Saleh , E. M. Elfekky , and A. M. Elmahmoudy , “CHA2DS2 VASc Score and Brachial Artery Flow ‐ Mediated Dilation as Predictors for No ‐ Reflow Phenomenon in Patients with ST ‐ Segment Elevation Myocardial Infarction Undergoing Primary Percutaneous Coronary Intervention,” Egyptian Heart Journal 74 (2022): 13. 10.1186/s43044-022-00249-x PMC8866621 35195795

[27] F. Mirbolouk , M. Gholipour , A. Salari , et al., “CHA2DS2 ‐ VASc Score Predict No ‐ Reflow Phenomenon in Primary Percutaneous Coronary Intervention,” Journal of Cardiovascular and Thoracic Research 10 (2018): 46–52. 29707178 10.15171/jcvtr.2018.08 PMC5913693

[28] G. Ipek , T. Onuk , M. B. Karatas , et al., “CHA2DS2 ‐ VASc Score Is a Predictor of No ‐ Reflow in Patients with ST ‐ Segment Elevation Myocardial Infarction Who Underwent Primary Percutaneous Intervention,” Angiology 67 (2016): 840–845. 26685178 10.1177/0003319715622844

[29] A. H. Shaikh , R. Kumar , A. Ammar , et al., “CHA2DS2 ‐ VASc Score, a Simple Clinical Tool for Early Prediction of No ‐ Reflow Phenomenon in Patients Undergoing Emergency Percutaneous Coronary Revascularization,” Journal of Cardiovascular and Thoracic Research 14 (2022): 122–127. 35935384 10.34172/jcvtr.2022.19 PMC9339737

[30] A. M. Nicolau , P. G. Silva , H. P. G. Mejia , et al., “Molecular Mechanisms of Microvascular Obstruction and Dysfunction in Percutaneous Coronary Interventions: From Pathophysiology to Therapeutics — A Comprehensive Review,” International Journal of Molecular Sciences 26 (2025): 6835. 40725082 10.3390/ijms26146835 PMC12295318

[31] S. A. Sabe , J. Feng , F. W. Sellke , and M. R. Abid , “Mechanisms and Clinical Implications of Endothelium ‐ Dependent Vasomotor Dysfunction in Coronary Microvasculature,” American Journal of Physiology ‐ Heart and Circulatory Physiology 322 (2022): H819–H841. 35333122 10.1152/ajpheart.00603.2021 PMC9018047

[32] A. Agarwal , R. Patel , and O. K. Khalique , “Coronary Microvascular Dysfunction: Bridging the Diagnosis ‐ Treatment Divide in Women with INOCA ‐ A Review,” Journal of Clinical Medicine 14 (2025): 6054. 40943815 10.3390/jcm14176054 PMC12429603

[33] K. S. Thomas , D. M. Puthooran , S. Edpuganti , A. L. Reddem , A. Jose , and S. S. M. Akula , “Reperfusion Injury in STEMI: A Double ‐ Edged Sword,” Egyptian Heart Journal 77 (2025): 83. 10.1186/s43044-025-00683-7 PMC12413384 40911117

[34] E. Cenko , J. Yoon , M. Bergami , et al., “Coronary Revascularization and Sex Differences in Cardiovascular Mortality After Myocardial Infarction in 12 High and Middle ‐ Income European Countries,” European Heart Journal — Quality of Care and Clinical Outcomes 11 (2025): 719–729. 38714331 10.1093/ehjqcco/qcae035 PMC12445642

[35] L. P. Dawson , M. Rashid , D. T. Dinh , et al., “No ‐ Reflow Prediction in Acute Coronary Syndrome During Percutaneous Coronary Intervention: The NORPACS Risk Score,” Circulation. Cardiovascular Interventions 17 (2024): e013738. 38487882 10.1161/CIRCINTERVENTIONS.123.013738

[36] M. Sawano , Y. Lu , C. Caraballo , et al., “Sex Difference in Outcomes of Acute Myocardial Infarction in Young Patients,” Journal of the American College of Cardiology 81 (2023): 1797–1806. 37137590 10.1016/j.jacc.2023.03.383

[37] M. Zachura , K. Wilczek , J. Kurzawski , M. Gierlotka , M. Gąsior , and M. Sadowski , “Gender ‐ Related Differences in Men and Women with ST ‐ Segment Elevation Myocardial Infarction and Incomplete Infarct ‐ Related Artery Flow Restoration: A Multicenter National Registry,” Advances in Interventional Cardiology 14 (2018): 356–362. 30603025 10.5114/aic.2018.79865 PMC6309832

[38] M. Zachura , M. Sadowski , J. Kurzawski , K. Piątek , and M. Gąsior , “Heterogeneity of the No ‐ Reflow Group After Primary Percutaneous Coronary Intervention Due to ST ‐ Segment Elevation Myocardial Infarction: Are There Sex Differences?,” Cardiovascular Revascularization Medicine 37 (2022): 97–101. 34167912 10.1016/j.carrev.2021.06.014

[39] L. Dong ‐ bao , H. Qi , L. Zhi , W. Shan , and J. Wei ‐ Ying , “Predictors and Short ‐ Term Prognosis of Angiographically Detected Distal Embolization After Emergency Percutaneous Coronary Intervention for ST ‐ Elevation Acute Myocardial Infarction,” Clinical Research in Cardiology 98 (2009): 773–779. 19730924 10.1007/s00392-009-0066-5

[40] R. Kumar , K. A. Khan , J. A. Shah , et al., “Quantification Of Thrombus Burden as an Independent Predictor of Intra ‐ Procedural No ‐ Reflow in Patients With ST ‐ Segment Elevation Myocardial Infarction Undergoing Primary Percutaneous Coronary Revascularization,” Journal of Ayub Medical College, Abbottabad 34 (2022): 288–294. 10.55519/JAMC-02-9698 35576288

[41] H. R. Reynolds , C. N. Bairey Merz , C. Berry , et al., “Coronary Arterial Function and Disease in Women With No Obstructive Coronary Arteries,” Circulation Research 130 (2022): 529–551. 35175840 10.1161/CIRCRESAHA.121.319892 PMC8911308

[42] M. Galli , H. A. W. Van't , and B. J. M. Ten , “Sex ‐ Related Variations in Platelet Reactivity in Presence or Absence of Antiplatelet Therapy,” European Heart Journal — Cardiovascular Pharmacotherapy 11 (2025): 56–65. 10.1093/ehjcvp/pvaf034 PMC12450592 40366913

[43] D. Kinoshita , K. Suzuki , H. Yuki , et al., “Sex ‐ Specific Association Between Perivascular Inflammation and Plaque Vulnerability,” Circulation. Cardiovascular Imaging 17 (2024): e016178. 38377234 10.1161/CIRCIMAGING.123.016178

[44] A. Nakajima , T. Sugiyama , M. Araki , et al., “Plaque Rupture, Compared With Plaque Erosion, Is Associated With a Higher Level of Pancoronary Inflammation,” JACC: Cardiovascular Imaging 15 (2022): 828–839. 34876381 10.1016/j.jcmg.2021.10.014 PMC12918542

[45] D. Fujimoto , D. Kinoshita , K. Suzuki , et al., “Relationship Between Calcified Plaque Burden, Vascular Inflammation, and Plaque Vulnerability in Patients with Coronary Atherosclerosis,” JACC: Cardiovascular Imaging 17 (2024): 1214–1224. 39243232 10.1016/j.jcmg.2024.07.013

[46] A. Gopal , S. Reddy , G. Kidess , et al., “Sex Disparities in STEMI Pathophysiology: Presentation, Treatments, and Outcomes,” Global Cardiology Science and Practice 2026 (2026): e202601. 10.21542/gcsp.2026.1 PMC13070267 41978757

[47] H. Yuki , T. Sugiyama , K. Suzuki , et al., “Coronary Inflammation and Plaque Vulnerability: A Coronary Computed Tomography and Optical Coherence Tomography Study,” Circulation. Cardiovascular Imaging 16 (2023): e014959. 36866660 10.1161/CIRCIMAGING.122.014959

