# Supplementary material for: Predictive Ability of the CHA2DS2‐VA Score for No‐Reflow Phenomenon in ST‐Elevation Myocardial Infarction Patients Undergoing Primary PCI
Source: Clin Cardiol. 2026 Aug 5;49(8):e70439. doi: 10.1002/clc.70439 (PMC13439631; doi:10.1002/clc.70439)
Supplement: Supplementary file 1 [file CLC-49-e70439-s001.docx]

**Supplementary Table 1. Predictive performance of the CHA_2_DS_2_-VA score alone and the full combined model for no-reflow phenomenon in STEMI patients undergoing primary PCI**

| **Variable** | **Overall (n = 725)** | **Female (n = 175)** | **Male (n = 550)** | **Sex × CV score interaction *p*** |
| --- | --- | --- | --- | --- |
| Model 1 AUC (95% CI) | 0.613 (0.547–0.679) | 0.673 (0.566–0.780) | 0.569 (0.486–0.651) | — |
| Model 1 *P* value† | 0.001 | 0.002 | 0.099 | — |
| Model 2 AUC (95% CI) | 0.689 (0.631–0.747) | 0.682 (0.572–0.792) | 0.721 (0.652–0.791) | — |
| Model 2 *P* value† | <0.001 | 0.001 | <0.001 | — |
| AUC difference (Model 2 − Model 1) | +0.076 | +0.009 | +0.152 | — |
| DeLong Z value | 2.806 | 0.675 | 3.256 | — |
| DeLong *P* value‡ | 0.005* | 0.500 | 0.001* | 0.017* |

* p-values < 0.05.

† *P* value indicates the statistical significance of AUC vs. 0.5 (reference line).

‡ DeLong test for comparison between Model 2 and Model 1.

Model 1 = CHA_2_DS_2_-VA score alone; Model 2 = CHA_2_DS_2_-VA score combined with sex, hemoglobin, platelet count, lymphocyte count, and number of stents. *AUC* = area under the ROC curve; *CI* = confidence interval.
